# Supplementary material for: Large near-term projected snowpack loss over the western United States
Source: Nat Commun. 2017 Apr 18;8:14996. doi: 10.1038/ncomms14996 (PMC5399290; doi:10.1038/ncomms14996)
Supplement: Supplementary Information — Supplementary Figures and Supplementary Table [file ncomms14996-s1.pdf]

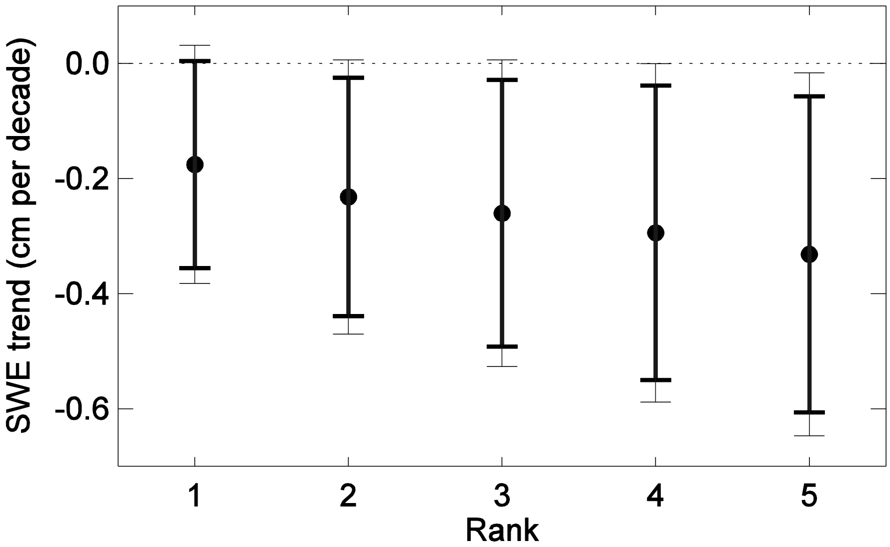

Supplementary Figure 1 | Trend in snow telemetry snow water equivalent. Trends are based monthly-mean averages over 354 stations over the Western United States over the snow season from January to May. A rank of 5 corresponds to the month of annual maximum snow water equivalent (SWE_{max}) which typically occurs in April. A rank of 4 corresponds to the month with the next largest value of SWE_{max}, and so on for the lower ranks. All stations are at elevations greater than 1500 meters and trends are computed from 1982 to 2010. The thick error bars are 15-85% uncertainty ranges, The thin error bars are 10-90% uncertainty ranges.

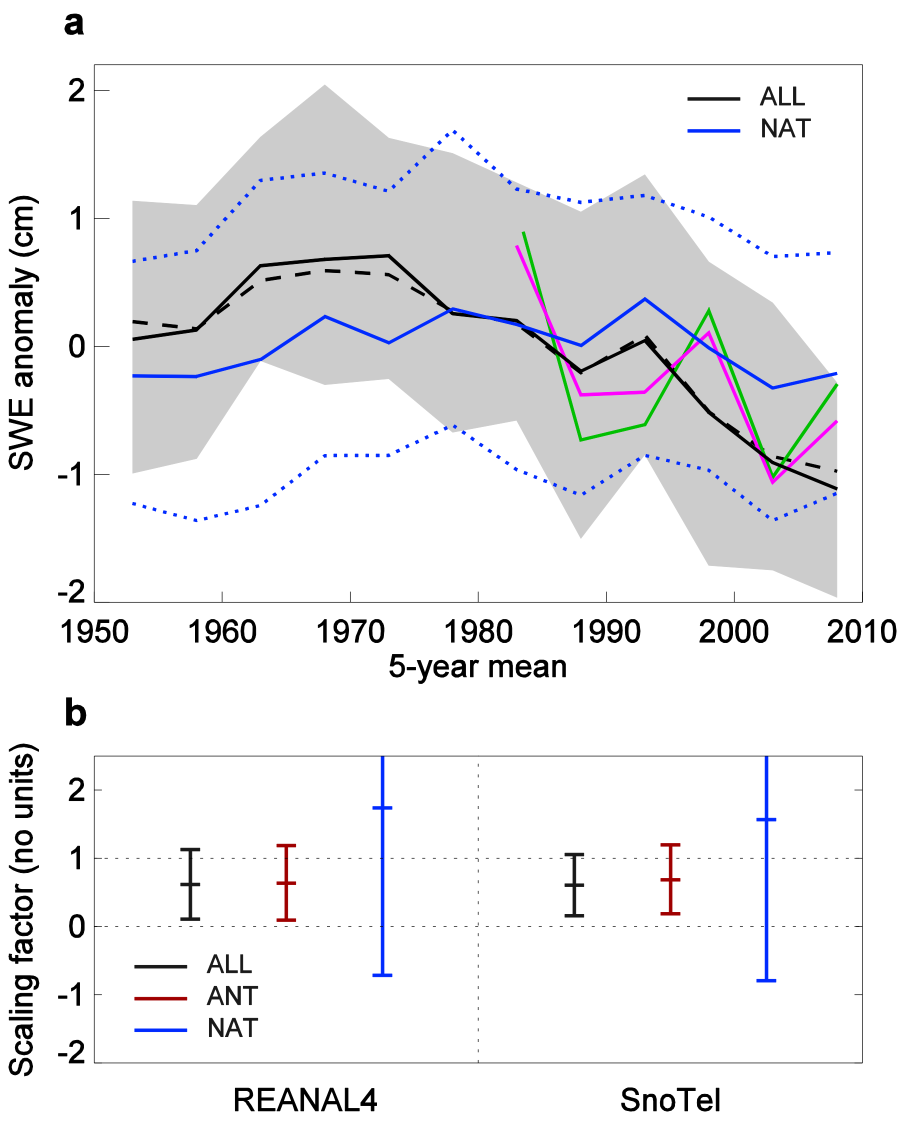

Supplementary Figure 2 | Anomaly of annual maximum snow water equivalent and scaling factor β. **a**, Anomaly in non-overlapping 5-year averages of annual maximum snow water equivalent (SWE_{max}) at elevations greater than 1000 meters. Solid black is ALL ensemble-mean and grey is 10-90% range (based on 50 ALL realizations). Solid blue line is NAT ensemble mean and dashed blue lines indicate the 10% and 90% values. The ALL and NAT curves are from CanESM2. The dashed black curve is from the 35-member set of CanRCM4 simulations with ALL forcing. Pink denotes the average of four reanalyses and green is the SnoTel observations. **b**, Scaling factor and 5–95% uncertainty range estimated from application of an optimal fingerprint method to SnoTel observations, reanalyses, and CanESM2 simulation output. Scaling factors greater than zero and consistent with one indicate that a model-predicted SWE_{max} signal has been detected in observations and attributed to the imposed forcing changes in the ALL or NAT simulations.

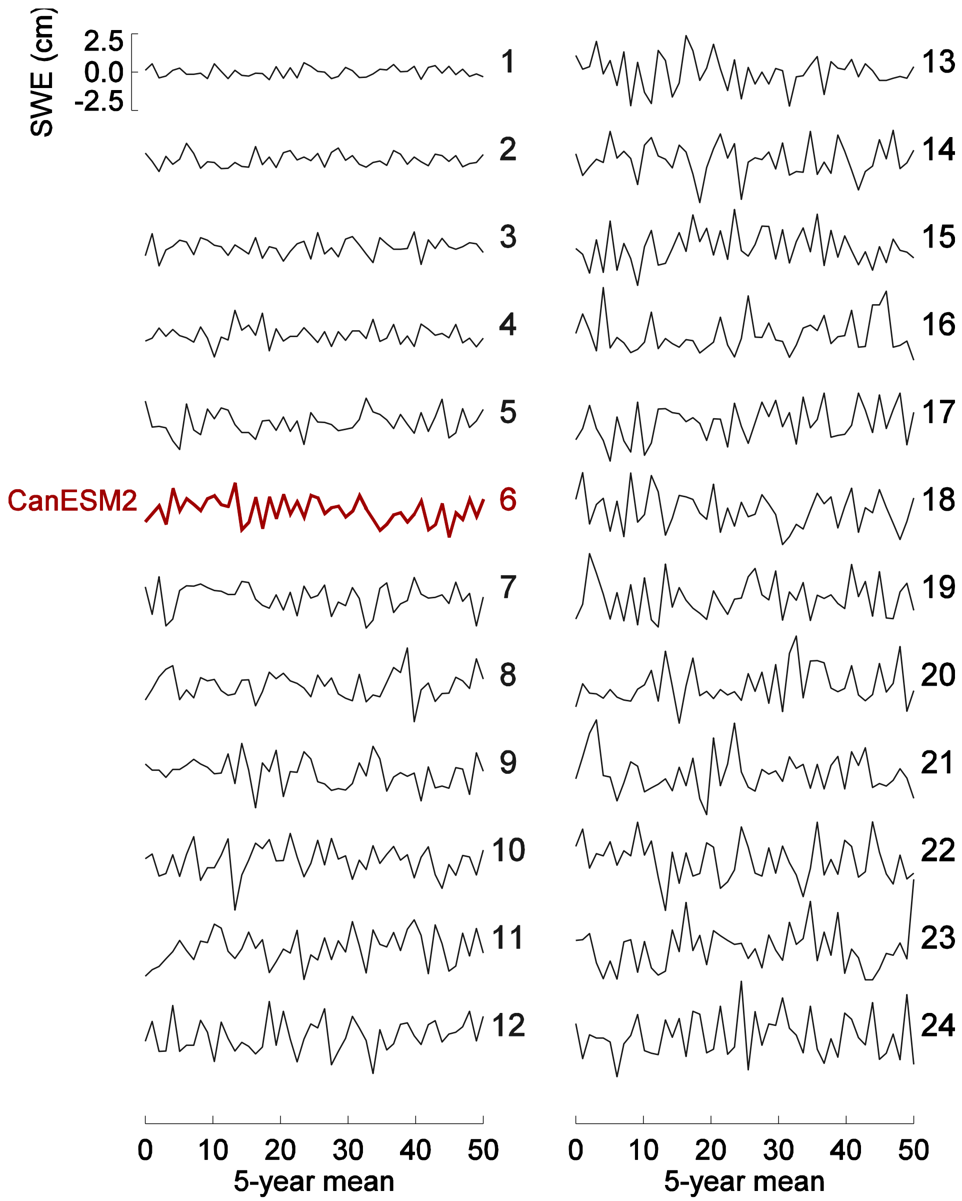

Supplementary Figure 3 | Anomaly of annual maximum snow water equivalent. Annual maximum snow water equivalent (SWE_{max}) anomalies in individual CMIP5 pre-industrial control model simulations (Supplementary Table 1) ranked by standard deviation. 5-year non-overlapping means are shown. Anomalies are relative to the long-term climatologies.

	piControl	Representative Concentration Pathway (RCP)		
		2.6	4.5	8.5
1	bcc-csm1-1-m	bcc-csm1-1	ACCESS1-0	ACCESS1-0
2	bcc-csm1-1	bcc-csm1-1-m	ACCESS1-3	ACCESS1-3
3	BNU-ESM	BNU-ESM	bcc-csm1-1	bcc-csm1-1
4	CanESM2	CanESM2	bcc-csm1-1-m	bcc-csm1-1-m
5	CCSM4	CCSM4	BNU-ESM	BNU-ESM
6	CESM1-CAM5	CESM1-CAM5	CanESM2	CanESM2
7	CNRM-CM5	CNRM-CM5	CCSM4	CCSM4
8	CSIRO-Mk3-6-0	CSIRO-Mk3-6-0	CESM1-BGC	CESM1-BGC
9	FGOALS-g2	FGOALS-g2	CESM1-CAM5	CESM1-CAM5
10	FIO-ESM	FIO-ESM	CMCC-CM	CMCC-CM
11	GFDL-CM3	GFDL-CM3	CMCC-CMS	CMCC-CMS
12	GFDL-ESM2G	GFDL-ESM2G	CNRM-CM5	CNRM-CM5
13	GFDL-ESM2M	GFDL-ESM2M	CSIRO-Mk3-6-0	CSIRO-Mk3-6-0
14	GISS-E2-H	GISS-E2-H	FGOALS-g2	FGOALS-g2
15	GISS-E2-R	GISS-E2-R	FIO-ESM	FIO-ESM
16	HadGEM2-ES	HadGEM2-AO	GFDL-CM3	GFDL-CM3
17	MIROC5	HadGEM2-ES	GFDL-ESM2G	GFDL-ESM2G
18	MIROC-ESM-CHEM	MIROC5	GFDL-ESM2M	GFDL-ESM2M
19	MIROC-ESM	MIROC-ESM-CHEM	GISS-E2-H-CC	GISS-E2-H-CC
20	MPI-ESM-LR	MIROC-ESM	GISS-E2-H	GISS-E2-H
21	MPI-ESM-MR	MPI-ESM-LR	GISS-E2-R-CC	GISS-E2-R-CC
22	MRI-CGCM3	MPI-ESM-MR	GISS-E2-R	GISS-E2-R
23	NorESM1-ME	MRI-CGCM3	HadGEM2-AO	HadGEM2-AO
24	NorESM1-M	NorESM1-ME	HadGEM2-CC	HadGEM2-CC
25	-	NorESM1-M	HadGEM2-ES	inmcm4
26	-	-	inmcm4	MIROC5
27	-	-	MIROC5	MIROC-ESM-CHEM
28	-	-	MIROC-ESM-CHEM	MIROC-ESM
29	-	-	MIROC-ESM	MPI-ESM-LR
30	-	-	MPI-ESM-LR	MPI-ESM-MR
31	-	-	MPI-ESM-MR	MRI-CGCM3
32	-	-	MRI-CGCM3	MRI-ESM1
33	-	-	NorESM1-ME	NorESM1-ME
34	-	-	NorESM1-M	NorESM1-M

Supplementary Table 1 | CMIP5 model simulations used in this study. The piControl refers to simulations without any external forcing changes. The representative concentration pathways reflect a range of emission scenarios from low, in the case of RCP2.6, to high in the case of RCP8.5.
